# An Online Calculator to Better Understand the Impact of False-Negative COVID-19 Polymerase Chain Reaction Test Results in the Context of Anesthesia Providers

**DOI:** 10.2196/26316

**Published:** 2021-04-14

**Authors:** Sean Runnels, John Frederick Pearson, Jon Dean Samuels, Rohan Kirit Panchamia

**Affiliations:** 1 Department of Anesthesiology University of Utah School of Medicine Salt Lake City, UT United States; 2 Department of Anesthesiology Weill Cornell Medical College New York, NY United States

**Keywords:** COVID-19, testing, false-negative rate, calculator, provider exposure, airway management, anesthesia, exposure, false negative, risk, transmission, infectious disease

## Abstract

What does the COVID-19 false-negative exposure problem mean in the context of a local anesthesia practice? We present a customizable online calculator designed to quantify and better understand individual and aggregate provider exposure risk.

Recently, Van Zundert et al [1] provided an excellent summary of the state of affairs concerning airway management and the Coronavirus Disease (COVID-19). One piece of the risk puzzle is a better understanding of the risk anesthesia providers face during the pandemic. A joint statement by the American Society of Anesthesiologists and the Anesthesia Patient Safety Foundation references a Centers for Disease Control and Prevention document recommending that patients scheduled for surgery should be screened for SARS-CoV-2 by polymerase chain reaction (PCR) testing, and if negative, the operating room staff can perform the surgery using only contact and droplet precautions [2,3].

The low sensitivity of SARS-CoV-2 PCR testing can lead to a high rate of false negatives [4,5]. These false-negative results—patients who are infected but test negative—may be most consequential to operating room staff, especially if donned in protective gear recommended for droplet precautions and not in gear recommended for aerosolizing procedures in COVID-19–positive patients. This is especially important as detection of the virus is unlikely prior to symptom onset [6].

Appreciating the true false-negative rate is an important start in determining provider-specific risk. An excellent online calculator is available to illustrate the impact of test sensitivity and pretest probability on the rate of false negatives [7]. We recommend this as a resource that may enhance one’s understanding of this issue in general. The obvious next step is to ask, “What does the false-negative rate mean in the local context of anesthesia to an individual provider or a group practice?” Perhaps a better way to state this is, “What does it mean to me and my practice?”

To allow dynamic, contextualized, and accessible understanding of the magnitude of risk posed by the false-negative problem in the context of an anesthesia provider, we have developed an online COVID-19 false-negative exposure risk calculator specifically for anesthesia providers. The Runnels & Pearson online calculator includes variable inputs of (1) SARS-CoV-2 prevalence, (2) PCR test sensitivity (estimated at 70%), (3) airways managed per day by an individual provider, and (4) number of airways managed by group per day. Each of these inputs is customizable, allowing inputs to reflect current local conditions or even model past or potential future scenarios. The calculator can be accessed online [8]. Calculated statistical outputs are (1) the false-negative rate; (2) cases performed per false-negative encounter; (3) individual provider workdays per false-negative exposure; and (4) number of providers encountering a false negative per day, week, and month. The University of Utah tests all patients within 3 days prior to elective surgery. On November 15, 2020, prevalence was 1.5% (49,575 active cases/3,280,000 people) [9]. Outputs are displayed for sensitivities of 70% and 90% in Figures 1 and 2.

**Figure 1:**
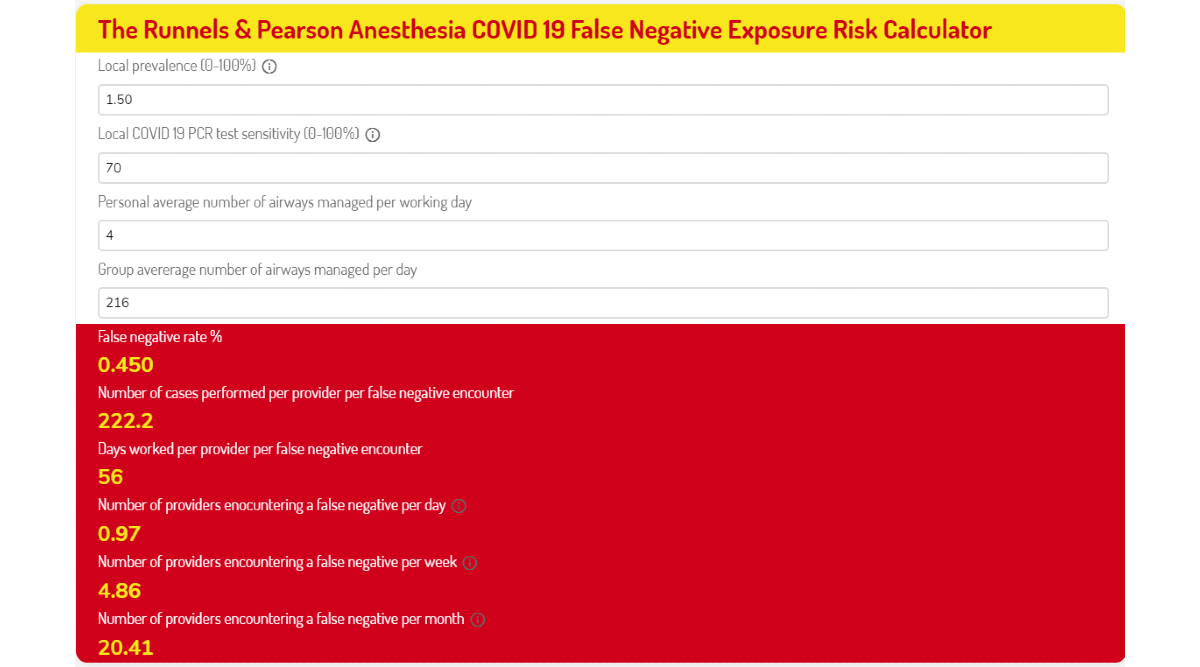
False-negative exposure risk calculator outputs for the University of Utah Department of Anesthesiology for November 15, 2020, at a COVID-19 test sensitivity of 70%. PCR: polymerase chain reaction.

**Figure 2:**
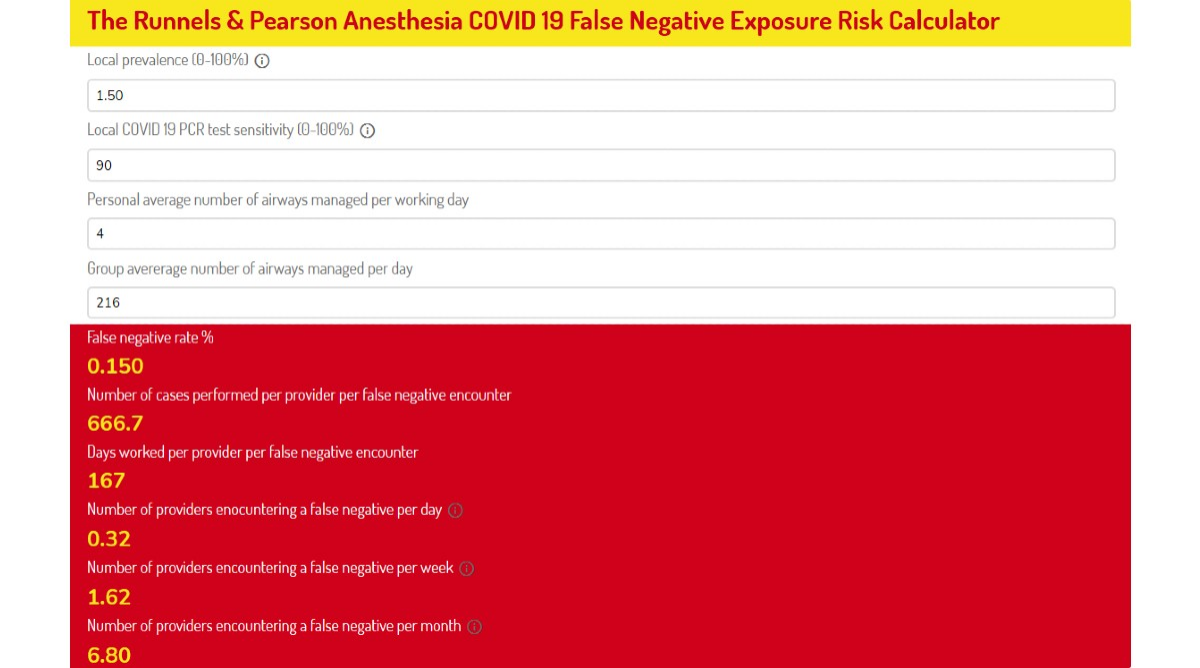
False-negative exposure risk calculator outputs for the University of Utah Department of Anesthesiology for November 15, 2020, at a COVID-19 test sensitivity of 90%. PCR: polymerase chain reaction.

Our goal is to create indices that have real meaning to providers and decision makers across disparate health care systems. This risk calculator can offer real-time, contextualized information that may offer part of a solution to the conundrum of uniform guidelines for heterogeneous risk. Perhaps guidelines of the future may be based on quantifiable risk thresholds, allowing guidelines to better fit the local situation on the ground. In addition, the Runnels & Pearson calculator may be used as a retrospective research tool to better understand how individual hospital or system guidelines concerning personal protective equipment (PPE) were made. For instance, a timeline comparing anesthesia provider risk and PPE guideline issuance might help us understand if these guidelines were data driven in nature.

Care must be taken to ensure that inputs into this calculator accurately reflect the data on the ground. We make no recommendations about sources for data inputs. Even with imprecise data inputs, this tool may be useful in generating a general understanding of risk in the context of anesthesia and operating rooms. We believe a general understanding can help facilitate better policy, guidelines, and allocation of resources in the service of improving the safety of patients and providers.

## Abbreviations

PCR: polymerase chain reaction
PPE: personal protective equipment
